# Too poor to science: How wealth determines who succeeds in STEM

**DOI:** 10.1371/journal.pbio.3003243

**Published:** 2025-06-23

**Authors:** Craig R. McClain

**Affiliations:** Department of Biology, University of Louisiana at Lafayette, Lafayette, Louisiana, United States of America

## Abstract

From student to researcher, a career in science can come with a high price tag. This Perspective explores how persistent financial barriers limit who can succeed in science, revealing how wealth shapes opportunity in STEM, and proposes structural changes to support equity and inclusion.

## Teaser

STEM is profoundly shaped by wealth, yet financial privilege is rarely acknowledged in discussions of diversity and inclusion. Wealth influences participation, persistence, and recognition in science, affecting not just who enters the pipeline but also who can afford to stay.

Behind the pursuit of scientific discovery lies a less-visible truth: a career in science, technology, engineering, and mathematics (STEM) is economically unsustainable, if not entirely unfeasible, for many. The myth of meritocracy in science collapses under the financial sacrifices expected at every career stage. From unpaid internships and self-funded conferences to underpaid graduate and postdoctoral positions, the hidden costs of ‘doing science’ are profound. These burdens disproportionately exclude those without generational wealth, compounded by race and gender. As federal funding declines and diversity, equity, and inclusion (DEI) initiatives are stripped away in the US, financial divides are widening into chasms, making science less accessible, equitable, and innovative. Ignoring these economic realities risks endangering the future of STEM.

Financial barriers emerge early, restricting students’ ability to build academic capital needed for STEM success. Attending a well-resourced and funded high school provides distinct advantages in terms of STEM exposure, preparation, and eventual access to competitive postgraduate programs [1]. Home resources also matter; students with limited access to computers at home consistently perform worse academically [2]. After-school programs improve academic outcomes but costs and transport often exclude students from low-income backgrounds [3]. Summer camps and enrichment opportunities offer vital development [4,5] but are often prohibitively expensive [6], worsening disparities. Many students from low-income backgrounds work during high school to support families, which negatively affects grades, test scores, and long-term goals, even at low weekly work hours [7].

Undergraduate students face additional financial barriers that hinder entry and success in STEM careers [8]. Attending a prestigious (often costly) college or university improves STEM outcomes through their superior resources, faculty, and networks. Many STEM degrees require additional lab fees, fieldwork and fieldtrip expenses, and costly elective (but essential) activities, like study abroad programs and professional development opportunities [8]. For students studying STEM, gaining research experience is vital for future success [9]. Although paid opportunities are available, they are extremely competitive due to limited numbers and high demand; alternatively, unpaid internships and research opportunities abound. Paid or not, these research experiences rarely offer compensation on par with other job opportunities and are forfeited by students who need an income [8]. The student loan crisis worsens disparities, forcing marginalized students into debt that harms long-term financial stability and careers [10]. Students with loan debt are less likely to enroll in postgraduate studies and student loan debt is associated with lower net worth, home equity value, and asset accumulation, impacting future stages of the STEM career [10]. The compounded effect of these STEM-specific financial barriers, along with general costs like textbooks, housing, and transportation, creates a significant disadvantage for students from a low-income background, making success in STEM a challenge.

Postgraduate studies bring increasing financial burdens that are frequently ignored or seen as ‘part of the process’. Many students receive teaching or research stipends, but in the US, these are modest and often below local living costs. Only 2 of 225 US biology PhD programs assessed offer annual salaries meeting basic living wages (https://rhettrautsaw.app/shiny/BiologyPhDStipends/). This salary insufficiency is often exacerbated by the unavailability of summer stipends. Postgraduate students are also expected to shoulder the costs of conference travel, professional memberships, software licenses, and research supplies, many of which are not fully reimbursed or incur long delays for reimbursement. For field-based disciplines, travel, equipment, and data collection expenses can be especially costly. Health care, childcare, and housing insecurity further compound the economic strain, particularly for students from low-income backgrounds or without familial financial support. These cumulative financial pressures and instability can restrict students’ ability to fully engage in research, limiting their agency for career development [11], unlike their wealthier counterparts.

Financial hurdles continue compounding throughout postdoctoral fellowships and beyond. Postdocs, many of whom hold temporary contracts with salaries well below the living wage, face chronic job insecurity, limited benefits, and recurring relocation costs with little institutional support. Even faculty may find themselves without grants and responsible for personally funding critical research activities, such as conference travel, journal publication, and purchasing lab supplies, when institutional funds fall short [12]. Additionally, salary compression, delayed promotion timelines, the expectation to ‘buy out’ portions of one’s salary through grants, and unpaid summer months for some faculty exacerbate long-term financial insecurity.

As faculty, those from lower-income backgrounds may also experience an acute dependence on their salaries and positions, making them hesitant to speak out, challenge norms, or draw attention to themselves. Pressured to increase earnings in compensation for low salaries earlier in their careers, many take on heavy service, teaching, or administrative loads that offer additional pay: choices that can come at the expense of research productivity and career advancement. Years of low income before becoming faculty can leave career scientists with little to no financial cushion and significant debt, delaying milestones like homeownership, starting a family, or saving for retirement. The cumulative financial strain of an academic STEM career often requires years, if not decades, for recovery. After a decade or more of surviving on poverty-level wages through postgraduate studies and postdocs, many scientists do not secure stable faculty positions until their late 30s or 40s; by then, years of missed retirement contributions and modest starting salaries deal a final financial blow, extending the economic precarity into retirement.

Despite these challenges, the institutionalized belief that being paid poorly is an acceptable part of career progression reflects a damaging narrative that does not align with the financial realities faced by many. This crisis does not just harm the scientific community’s growth but also worsens the financial struggles of scientists, leading to poor personal financial decisions, immense stress, and mental health challenges [13]. Many talented scientists, disillusioned by constant financial insecurity, leave academia altogether, resulting in a significant brain drain that deprives the field of diverse perspectives and innovation. Moreover, as STEM becomes increasingly exclusionary, wealthier individuals can afford a different STEM outcome, further entrenching privilege and widening the disparities within the academic system.

Real change is needed to address these systemic issues. First, academia must acknowledge this problem exists throughout the life of an academic. Even a small, early injection of funding, can lead to persistence in STEM [14]. Postgraduate students are more likely to persist in their programs with tuition reductions, load deferrals, and moderate educational debt [13]. Obviously, reinstating and protecting DEI funding for underrepresented groups is essential. Some additional recommendations include: increasing postgraduate stipends to meet local living wages; requiring paid research internships for undergraduate students to help alleviate financial stress early on; expanding funding and structural support for researchers; including increased salary support, particularly for those not able to secure summer salaries; internal funding pools to cover research and travel costs; and providing support for faculty to secure housing (e.g., housing bonuses or no-to-low-interest home loans). Additionally, journals and organizations can help reduce financial burdens by further waiving open-access and conference fees for financially vulnerable researchers. However, these financial solutions are complicated by universities’ limited and shrinking budgets, especially in locations with fewer resources, and the ongoing cuts to US federal funding.

Without more money, universities can shift priorities: reevaluate what counts as merit in hiring and promotion; foster collaborations and peer mentorships; expand access to in-house resources; and streamline bureaucratic processes. Institutions can offer grant-writing support, share equipment across departments, and build inclusive, no-cost professional networks. Programs, opportunities, and positions should disclose estimated out-of-pocket costs such as housing, travel, and unpaid time up front. We should encourage and value low-cost research approaches, such as local place-based studies, open-source tools, big data analyses, and open-access projects that reduce expensive travel or logistics. Open access and open science—including open data, tools, and publications—can help reduce financial barriers and increase equity. As a field, we must compile and share free or low-cost resources: fellowships, journals, databases, and support networks. Most importantly, we must shift the culture, rejecting the idea that financial struggle is a rite of passage and embracing equity as a shared responsibility.

## Abbreviations

DEI: diversity, equity, and inclusion
STEM: science, technology, engineering, and mathematics
